# Androgenic regulation of beta-defensins in the mouse epididymis

**DOI:** 10.1186/1477-7827-12-76

**Published:** 2014-08-07

**Authors:** Shuang-Gang Hu, Mei Zou, Guang-Xin Yao, Wu-Bin Ma, Qin-Ling Zhu, Xiang-Qi Li, Zi-Jiang Chen, Yun Sun

**Affiliations:** 1 Center for Reproductive Medicine, Renji Hospital, School of Medicine, Shanghai Jiaotong University, Shanghai Key Laboratory for Assisted Reproduction and Reproductive Genetics, Shanghai 200135, China; 2 Shanghai Key Laboratory for Molecular Andrology, State Key Laboratory of Molecular Biology, Institute of Biochemistry and Cell Biology, Shanghai Institutes for Biological Sciences, Chinese Academy of Sciences, Shanghai 200030, China

**Keywords:** Androgen, Androgen receptor, Epididymis, Beta-defensins

## Abstract

**Background:**

The majority of beta-defensin family members are exclusively expressed in the epididymis, and some members have been shown to play essential roles in sperm maturation and fertility in rats, mice and humans. Therefore, beta-defensins are hypothesized to be potential targets for contraception and infertility diagnosis and treatment. Clarifying the regulatory mechanisms for the expression of these genes is necessary. Androgen/androgen receptor (AR) signaling plays an important regulatory role in epididymal structure and function. However, very little is known about the androgenic regulation on the production and secretion of the epididymal beta-defensins.

**Methods:**

The expression of beta-defensins was detected by quantitative RT-PCR. The androgen dependence of beta-defensins was determined by bilateral orchiectomy and androgen supplementation. The androgen response elements (AREs) in the promoters of beta-defensins were identified using the MatInspector software. The binding of AR to AREs was assayed by ChIP-PCR/qPCR.

**Results:**

We demonstrated that 23 mouse caput epididymal beta-defensins were differentially regulated by androgen/androgen receptor. Six genes, Defb18, 19, 20, 39, 41, and 42, showed full regulation by androgens. Ten genes, Defb15, 30, 34, 37, 40, 45, 51, 52, 22 and Spag11a, were partially regulated by androgens. Defb15, 18, 19, 20, 30, 34, 37, 39, 41, 42, 22 and Spag11a were associated with androgen receptor binding sites in their promoter or intronic regions, indicating direct regulation of AR. Six genes, Defb1, 12, 13, 29, 35, and spag11b/c, exhibited an androgen-independent expression pattern. One gene, Defb25, was highly dependent on testicular factors rather on androgens.

**Conclusions:**

The present study provides novel insights into the mechanisms of androgen regulation on epididymal beta-defensins, enabling a better understanding of the function of beta-defensins in sperm maturation and fertility.

## Background

Beta-defensins are small cationic peptides that exhibit broad-spectrum antimicrobial properties and contribute to mucosal immune responses at epithelial sites. Recently, the complete genome sequences of different species and computational prediction and experimental verification have identified 30–50 novel beta-defensin genes in humans, rats and mice that are organized into gene clusters localized at specific chromosomes [1,2]. The mRNAs encoding the majority of beta-defensins are exclusively expressed in the epididymis [3,4]. Epididymal beta-defensins with antimicrobial activity [5-7] have been shown to be involved in epididymal innate immune protection; however, recent studies have indicated that several of these peptides could bind to the sperm surface and play novel roles in male reproductive physiology. Rat Bin1b, which binds to the sperm head, initiates sperm motility in immature sperm from the caput epididymidis by a mechanism dependent on calcium uptake [8]. Likewise, immunization with the Bin1b peptide induced the production of anti-Bin1b antibodies and resulted in reduced fertility in rats [9]. Defb15, which binds to the sperm acrosomal region and forms part of the sperm glycocalyx, is required for sperm motility and male fertility. The in vivo knockdown of the rat Defb15 gene by RNAi led to a considerable attenuation of sperm motility and fertility [7]. Studies that include target deletion of Defb15 have also demonstrated homozygous males with low motility sperm and a reduced fertility phenotype [10]. Recent studies have further enhanced our understanding of the role of beta-defensins in fertility and sterility. Zhou et al. reported that the homozygous deletion of a cluster of nine β-defensin genes (DefbΔ9) in mice resulted in male sterility [11]. Tollner et al. reported that a common mutation in the human defensin Defb126 causes reduced sperm penetration ability and is associated with subfertility [12].

Because of their important functions in sperm maturation and fertility, beta-defensins are receiving more attention and are hypothesized to be potential targets for diagnosing and treating infertility. Therefore, clarifying the regulation mechanisms on their expression is greatly required. However, up to now, very little is known about the regulation of the production and secretion of epididymal beta-defensins.

Androgen signaling plays an important regulatory role in epididymal structure and function. Many epididymal secretory proteins involved in sperm maturation have been identified as androgen-regulated proteins. The effects of androgen are mediated through the androgen receptor (AR), a ligand-inducible nuclear receptor that regulates the expression of target genes by binding to androgen response element (ARE) DNA [13]. The identification of the beta-defensin transcripts that are regulated by androgens might be important to help elucidate the process of sperm maturation. Several beta-defensins have been reported to be regulated by androgen in different species, including rats, monkeys, humans and mice. In mice, only five beta-defensins, including Spag11a (Bin1b), Defb20, 22, 41, and 42 [14-17], have been shown to be regulated by androgen. However, the previous study was unable to determine whether these beta-defensins are direct or indirect AR targets. Additionally, these previous studies were focused on the individual beta-defensin genes; a systematic profile of the androgenic regulation of the beta-defensin family is lacking.

In the epididymis, the majority of beta-defensins are abundantly expressed in the caput region, which expresses high levels of AR and proteins required for sperm maturation. In this study, we systematically investigated androgen regulation on the expression of beta-defensins in the mouse caput epididymidis. Additionally, we screened and identified the AR binding sites associated with some androgen-regulated beta-defensins, indicating direct AR regulation of these defensins.

## Methods

### Castration and androgen replacement

Adult 10-wk-old male C57BL/6 J mice were divided into the following three groups (six mice/group): an intact group (no castration), an Oil group (castration + oil) and a testosterone propionate (TP) group (castration + TP). The mice were castrated bilaterally under sodium pentobarbital anesthesia. After 7 days of recovery, all of the castrated mice were divided into two groups: the Oil group and the TP group. The Oil group was treated with a 100-μl injection of 90% sesame oil and 10% ethanol. The TP group was injected with 2.5 mg of TP dissolved in 90% sesame oil and 10% ethanol once every 24 h, for a total treatment of 5 mg TP over a 48-h period. The oil and TP were injected into the peritoneal cavity of the mice. All of the mice were killed 48 h post-injection, and the caput epididymides (not including the initial segment) from each group were removed for chromatin immunoprecipitation (ChIP) or real-time polymerase chain reaction (RT-PCR). All of the mouse treatments were repeated three times. The testosterone content of the serum samples was measured using a radioimmunoassay (RIA). The animal experiments were conducted by following a protocol approved by the Institute Animal Care Committee. The protocol conforms to internationally-accepted guidelines for the humane care and use of laboratory animals.

### ChIP

Three adult male C57BL/6 J mice from each group were sacrificed, and the caput epididymides were removed, pooled and finely minced in phosphate-buffered saline (PBS). After washing twice with PBS, the tissue debris was cross-linked in 1% formaldehyde at room temperature for 10 min. The debris was pelleted, washed and resuspended in PBS containing protease inhibitors. The debris was then disaggregated (maintaining the samples on ice) using a glass homogenizer with 50 strokes. The nuclei were collected and sonicated to yield 100-500-bp DNA fragments. The lysates were precleared with protein A beads (sc-2001, Santa Cruz, USA) at 4°C for 2 h before an overnight incubation with 4 μg of anti-AR antibody (H-280, sc-13062, Santa Cruz, USA) or normal rabbit IgG (sc-2027, Santa Cruz, USA). After 2 h of incubation with the protein A beads, the chromatin-antibody-beads complex was washed twice with low salt buffer, twice with high salt buffer, twice with lithium chloride buffer and three times with TE buffer. Then, the chromatin was eluted with elution buffer (1% SDS, 0.1 M NaHCO_3_) before the reversal of cross-linking with proteinase K (Invitrogen, Carlsbad, CA) at 65°C for 4 h. The DNA was purified by two rounds of phenol-chloroform extraction and ethanol precipitation and was resolved in an optimal volume of ddH_2_O.

### RNA extraction and quantitative RT-PCR

The total RNA was extracted from the mouse caput epididymidis (not including the initial segment) using TRIzol (Invitrogen, Grand Island, USA) according to the manufacturer’s instructions. One microgram of total RNA was used to synthesize the first-strand of cDNA with ReverTra Ace reverse transcriptase (Toyobo, Japan) and oligo dT under the conditions recommended by the manufacturer. Quantitative RT-PCR was performed using SYBR Green master mix (Toyobo, Japan) with a Rotor-Gene 3000 machine (Corbett Research, Sydney, Australia). The samples and standard curves were performed in triplicate, and the relative standard curve method was used to calculate the relative gene expression. A housekeeping gene, ribosomal protein S2, was included as the endogenous normalization control to adjust for unequal amounts of RNA. The primers for the defensin genes are listed in Additional file 1: Table S1.

### ChIP-PCR and ChIP-qPCR

ChIP assays were performed on mouse caput epididymides (n = 3) as described above. Primers were designed to amplify a 90- to 150-bp region surrounding the known binding peaks. DNA samples from the ChIP preparations were analyzed using conventional PCR or quantitative PCR with SYBR Green Master Mix (Toyobo). The primers are listed in Additional file 2: Table S2.

### Bioinformatics

Potential ARE search: The sequences of AR binding sites were scanned for potential ARE motifs using the MatInspector software (Genomatix Software GmbH, Germany) with the default settings. MatInspector utilizes MatBase Matrix Library 8.1, which is a comprehensive transcription factor binding site database that is frequently used to locate DNA sequences. Sequence logos for potential AREs were constructed using Weblogo (http://weblogo.berkeley.edu/logo.cgi) with the default settings.

Genomic mapping of ARBSs: The file containing unique ChIP-seq tags in the WIG format was uploaded to the UCSC genome browser on the mouse mm9 assembly (http://genome.ucsc.edu/cgi-bin/hgGateway) using “add custom tracks”. The distribution of ARBSs around AR target beta-defensin genes was displayed at optimal settings in “configure tracks and display”.

### Statistical analyses

Three replicates of all data were performed and used to determine statistical significance. All statistical analyses were carried out with SPSS13.0 (Chicago, IL, USA). Data are expressed as the mean ± SD. For determination of significant differences among groups, one-way analysis of variance was used. P < 0.05 was considered statistically significant in all the tests.

## Results

### Identification of beta-defensin genes expressed in the caput epididymidis

We focused our study on caput epididymidis and compiled 23 beta-defensin genes expressed in the caput region based on the literature [4,15,16,18,19]. These genes include Defb1, 12, 13, 15, 18, 19, 20, 22, 25, 29, 30, 34, 35, 37, 39, 40, 41, 42, 45, 51, and 52, Spag11a, and Spag11b/c. The NCBI Gene ID, the RefSeq accession number and the known aliases of these genes are listed in Table 1. Some mouse beta-defensin genes have other aliases or designations; for example, Defb41 is also known as Defb16, Defb19 is also known as Defb24, Defb45 is also known as Defb27, Defb42 is also known as Defb44, and Spag11a is also known as Ep2e, Bin1b or Spag11. In our study, only the official names provided by mouse genome informatics (MGI) were used.

**Table 1:** Gene ID, RefSeq accession number and known aliases of 23 beta-defensins

**Gene name**	**Gene ID**	**RefSeqaccession number**	**known aliases**
*defb1*	13214	NM_007843.3	AL590630.1, AW260221, BD-1
*defb12*	77674	NM_152802.3	9230103N16Rik, BD-12
*defb13*	246083	NM_139223.3	AL590619.7, BD-13
*defb15*	246082	NM_139222.3	AL590619.5, BD-15
*defb18*	654460	NM_001039123.1	BD-18, EG654460, mBD-18
*defb19*	246700	NM_145157.3	BD-19, Defb24, Tdl, mBD-19
*defb20*	319579	NM_176950.3	RP23-453 K8.4, BD-20, mBD-20
*defb22*	442835	NM_001002791.2	RP23-453 K8.5, 9230002F21Rik
*defb25*	654459	NM_001039122.1	RP23-35I8.8, BD-25, mBD-25
*defb29*	75400	NM_001001444.2	RP23-35I8.1, BD-29, mBD-29
*defb30*	73670	NM_001039566.2	BD-30, mBD-30
*defb34*	360211	NM_183035.1	BD-34
*defb35*	246084	NM_139224.1	BD-35
*defb37*	353320	NM_181683.2	BD-37
*defb39*	360214	NM_183038.2	BD-39
*defb40*	360217	NM_183039.3	BD-40
*defb41*	77673	NM_183124.3	Bd-41, Defb16
*defb42*	619548	NM_001034910.3	Defb44
*defb45*	433490	NM_001037752.2	Defb27;
*defb51*	100503992	NM_001025353.2	Gm6040, EG574083
*defb52*	100504014	NM_001177471.1	Gm15056,
*spag11a*	78128	NM_153115.1	Bin1b, EP2Q, EP2e, Spag11
*spag11b/c*	546038	NM_001039563.3	Ep2c/h; EG546038; Spag11c/h

### Androgen regulation of beta-defensin gene expression in the mouse caput epididymidis

**Figure 1:**
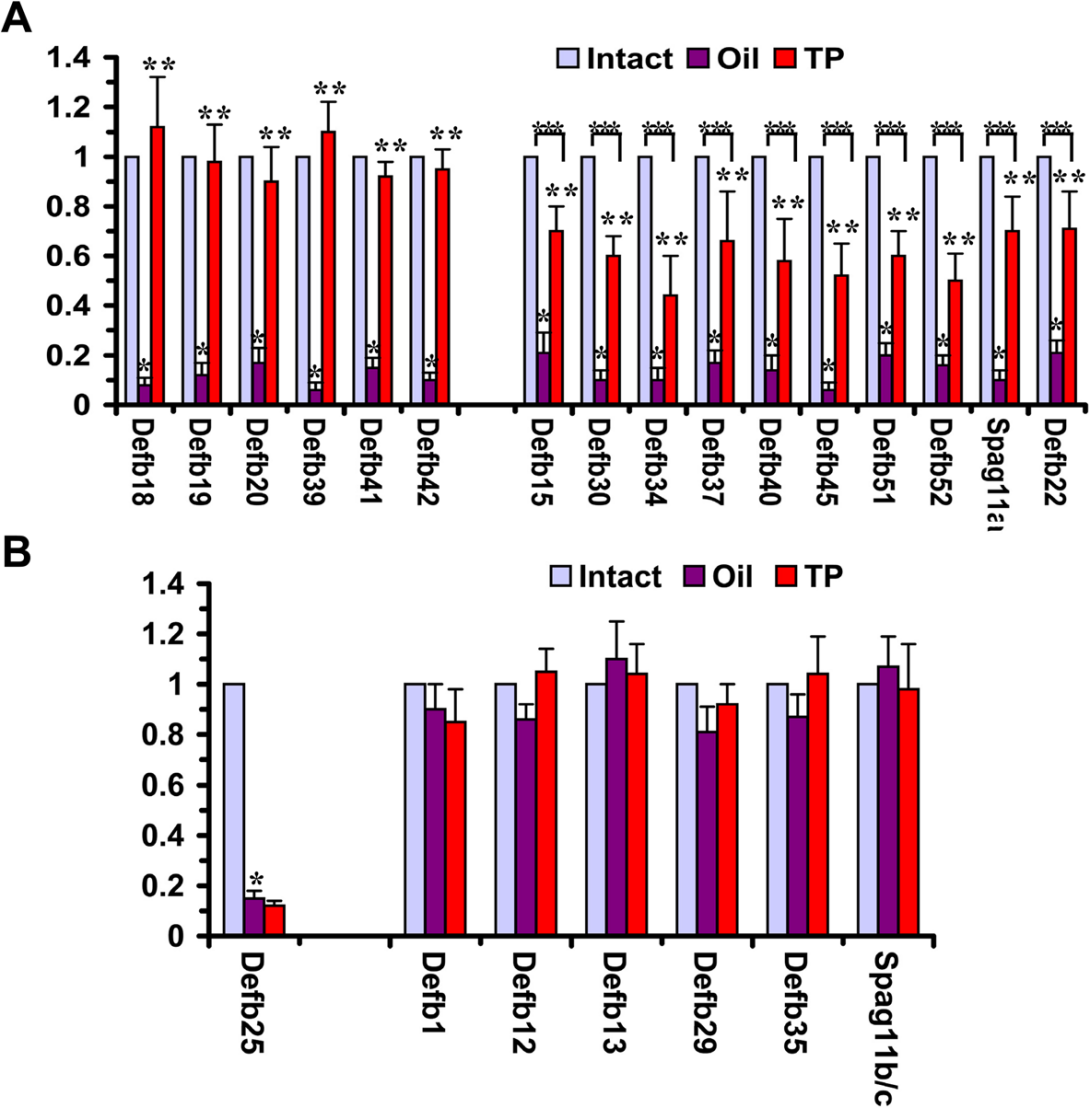
**The responsiveness of epididymal beta-defensins to androgen manipulation as revealed by quantitative RT-PCR.** RNA samples were prepared from the caput epididymides of intact mice (**Intact**) and mice castrated for 7 days and supplemented with oil (**Oil**) or testosterone propionate (**TP**). Different genes showed different responsiveness to androgen. **A)** The gene expression was fully or partially dependent on androgen. *: P < 0.01 Oil group versus Intact group; **: P < 0.05 TP group versus Oil group. ***: P < 0.05 TP group versus Intact group. **B)** The expression of Defb25 was dependent on testicular factors and not on androgen, whereas the expression of other genes was independent on testicular factors or androgen. *: P < 0.05 Oil group versus Intact group.

To investigate the transcriptional regulation of beta-defensin genes, we analyzed the expression of these genes in gonadectomized mice. We performed quantitative RT-PCR analyses using total RNAs isolated from the caput epididymides of intact mice and mice that had been bilaterally castrated, reared for 1 week and then treated with TP or oil. As shown in Figure 1A, the expression of 16 genes, including Defb15, 18, 19, 20, 30, 34, 37, 39, 40, 41, 42, 45, 51, 52, and 22 and spag11a, partially or completely disappeared after castration and oil treatment (p < 0.01), whereas after TP treatment, the mRNA level of these genes was significantly increased compared with the mRNA level after oil treatment (p < 0.05), indicating the androgenic regulation of the expression of these genes. The expression levels of 6 genes, Defb18, 19, 20, 39, 41, and 42, were increased and were approximately consistent with the levels observed in the intact mice after TP treatment (p > 0.05), indicating complete regulation by androgens. The expression levels of ten genes, Defb15, 30, 34, 37, 40, 45, 51, 52, and 22 and spag11a, were significantly lower after TP treatment compared with the levels in intact mice, indicating partial regulation by androgens (p < 0.05). Six genes, Defb1, 12, 13, 29, and 35 and spag11b/c, exhibited an androgen-independent expression pattern (Figure 1B). The expression levels of these genes did not change notably with castration and TP treatment, indicating that they are constitutively expressed in the epididymis, regardless of the presence of androgen or testicular factors. One gene, Defb25, was down-regulated by castration, and the expression was not increased after TP treatment, indicating that its expression was highly dependent on testicular factors rather than on androgens (Figure 1B).

### AR direct regulation on target beta-defensins

**Figure 2:**
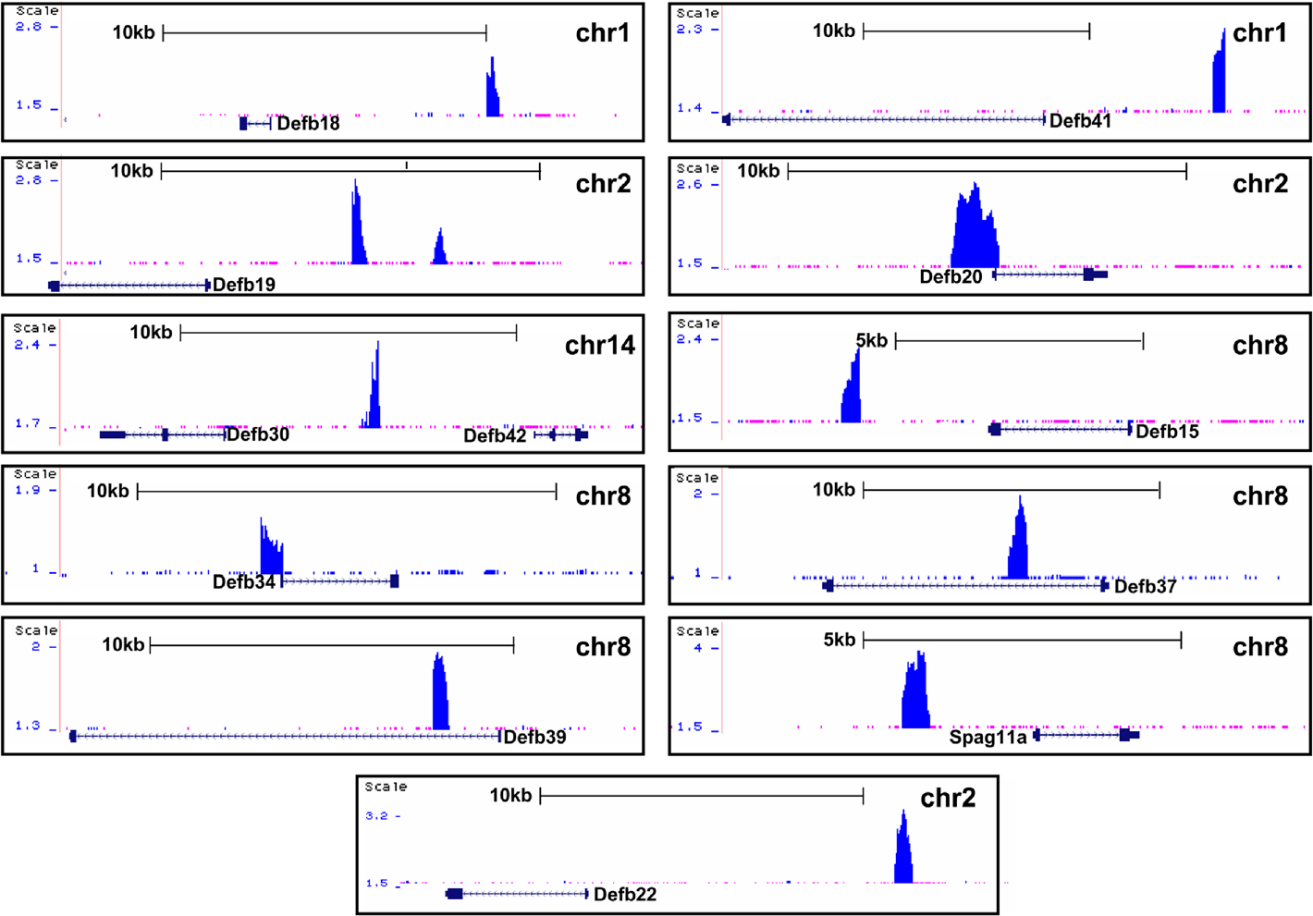
**Screenshots of the UCSC genome browser mm9 showing AR binding sites around the AR-target beta-defensin genes.** The figure was generated by uploading the file containing unique ChIP-seq tags in the WIG format to the UCSC genome browser. The upper blue track shows the tag density, and the AR-target beta-defensin genes are shown at the bottom. The black arrow indicates gene orientation. Sequence positions and other generic UCSC annotations were removed for clarity. One peak formed by a large amount of localized ChIP-seq tags represents an AR binding site. Defb19 was associated with two peaks, whereas Defb30 and Defb42 share one peak. Defb18, 34, 39, 41, 20, 15, 37, and 22 and Spag11a were associated with one peak respectively.

We previously reported the genome-wide androgen receptor binding sites (ARBSs) in the caput epididymidis using ChIP-seq [14]. Of 16 androgen regulated beta-defensins, 12 had one or more ARBSs within the region 10 kb upstream of the transcription start site to 5 kb downstream of the 3′-end of the gene. As shown in Figure 2 and Table 2, eleven defensin genes were associated with only one ARBS, whereas Defb19 was associated with two ARBSs. One ARBS was simultaneously associated with Defb30 and Defb42, implying the potential dual regulatory functions of this site. The ARBSs associated with Defb18, 19, 20, 30, 34, 41, 42, and 22 and Spag11a were located upstream of the cognate transcription start site, the ARBS associated with Defb15 was located in the downstream of the 3′-end, and the ARBSs associated with Defb37 and Defb39 were located in the intronic region.

**Table 2:** Characterization of ARBSs associated with androgen regulated epididymal beta-defensins

**Gene name**	**Chr**	**ARBS distance to TSS**	**ARBS location**	**Fold enrichment**	**Potential ARE?**	**Potential ARE distance to TSS**	**Potential ARE sequence**
*Spag11a*	chr8	−1914	upstream	113.6	Yes	−1844	CGGCAACTTTCTGTACTTT
						−1822	GAAGGACAGAAAGTCCTGT
						−1740	GATGGACACTTTGTCCTCT
*Defb15*	chr8	2842	downstream	49.3	Yes	2726	TCAGCACTTACTGTTCCTG
*Defb18*	chr1	−6911	upstream	36.9	Yes	−7011	GAATCTTTTTATGTTCTCT
						−7020	TATGTTCTCTATGTTCTGT
*Defb19*	chr2	−5979	upstream	32.9	Yes	−6075	ATCGATCGCTGTGTTCCCA
						−6106	TGGGAGCCACTTGTTCCTT
	chr2	−3900	upstream	27.2	Yes	−3970	ATGCTATAGTTTGTTCTCA
						−4012	TAGGAACAGCTTGGCCTGA
*Defb20*	chr2	−698	upstream	48.3	No		
*Defb30*	chr14	−4251	upstream	33.2	No		
*Defb34*	chr8	−222	upstream	21.3	Yes	−110	ATGGGGTGATTTGTTCTGA
*Defb37*	chr8	1659	intron	19.0	Yes	1609	AGGGAAAGCTGTGTTCTTG
						1555	GATCAAAGCCTTGTTCTTT
						1531	CAAGGACACTGTGTGCTGA
*Defb39*	chr8	1750	intron	40.9	Yes	1647	TGGGCACACAGTGGCCTTG
*Defb41*	chr1	−7576	upstream	63.6	Yes	−7633	CAAGAACACTCTGTGATAT
						−7692	CCAGAACAACATGTAACAT
*Defb42*	chr14	−4893	upstream	33.2	No		
*Defb22*	chr2	−9848	upstream	60.5	Yes	−9895	ACAGCATGCTGTGTTCTTC
						−9958	TTAGAACAACACGATCTCA

ARBS, androgen receptor binding site; TSS, transcription start site; ARE, androgen response element.

**Figure 3:**
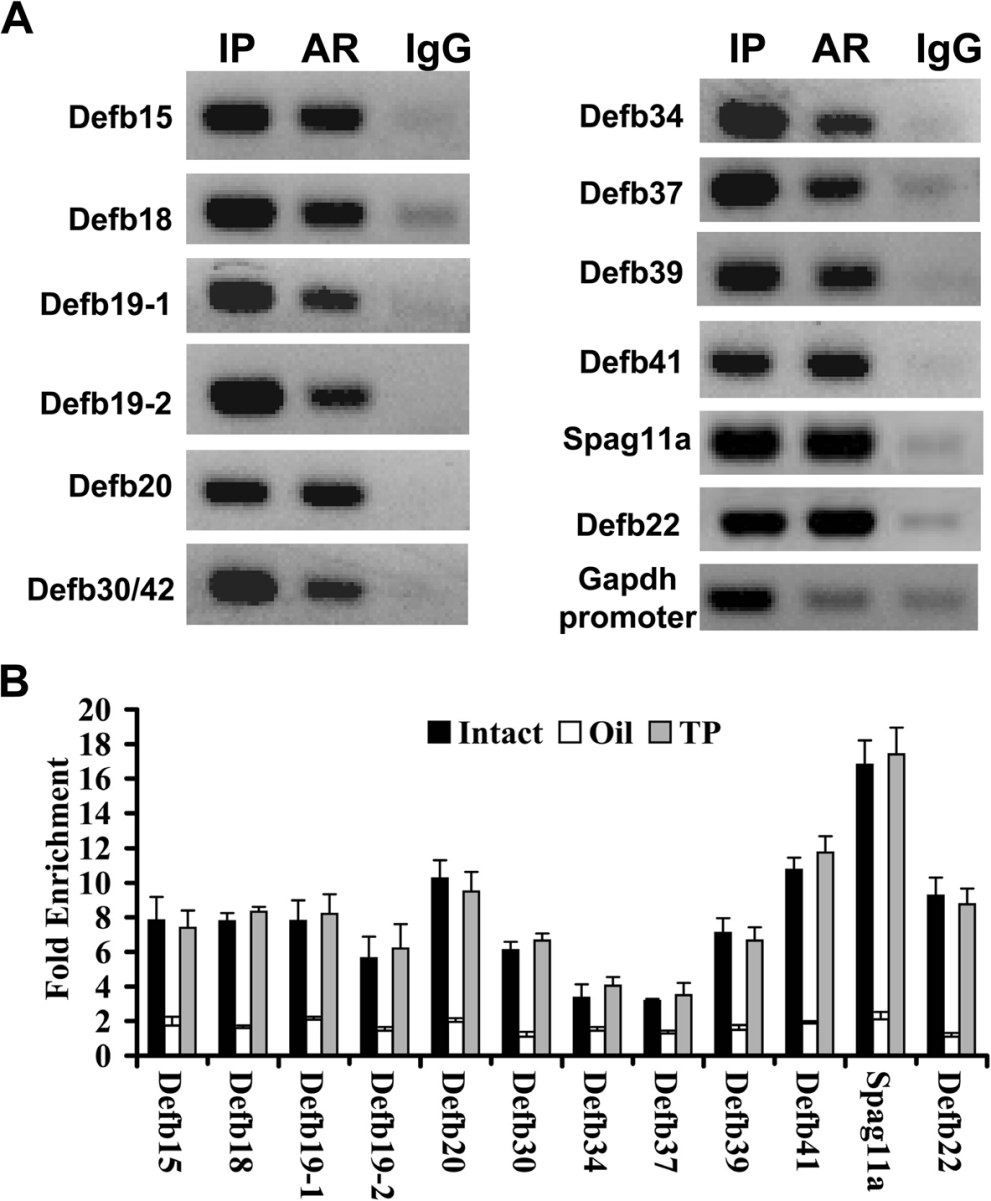
**AR occupancies on epididymal AR binding sites associated with androgen-regulated beta-defensins as revealed by ChIP-PCR (A) or ChIP-qPCR (B). A)** Input sample (**IP**) and ChIP samples of anti-AR antibody (**AR**) or normal IgG **(IgG**) were analyzed using PCR to confirm AR binding at eleven epididymal target loci. **B)** ChIP samples were prepared from the caput epididymidis of intact mice (**Intact**) and mice castrated for 7 days and supplemented with oil (**Oil**) or testosterone propionate (**TP**) for an additional 2 days. Enrichment folds were calculated using IgG enrichment as a control. The data are presented as the mean ± SD of three replicates.

The 12 ARBSs were searched for potential AREs. As shown in Table 2, the 10 ARBSs associated with Defb15, 18, 19, 34, 37, 39, 41, and 22 and Spag11a contained one or more potential AREs, whereas the 2 ARBSs associated with Defb20, 30 and 42 were not potential AREs. The sequence logo for the potential ARE motif overrepresented in the above-mentioned 10 ARBSs was generated based on sequence similarity. As shown in Additional file 3: Figure S1, the identified potential ARE motif from 10 ARBSs (*top panel*) was very similar to the established consensus canonical ARE in the MatBase database (*bottom panel*).To validate the ChIP-seq results, the ARBSs associated with androgen-regulated beta-defensins were confirmed by individual ChIP assays combined with conventional PCR (ChIP-PCR) or quantitative PCR (ChIP-qPCR). As shown in Figure 3A, all of the ARBSs were occupied by AR in the caput epididymidis under physiological conditions. Given that AR recruitment to target DNA is androgen-dependent, we assessed the androgen dependence of AR recruitment to ARBSs using ChIP-qPCR. AR binding was abolished after castration and recovered following androgen supplementation (Figure 3B), indicating that AR occupancy is androgen dependent.

## Discussion

The epididymis is an androgen-responsive organ responsible for sperm maturation and storage. Compared with other hormones and testicular factors, androgens play important regulatory roles in epididymal structure and function [20]. There is a high level of androgen receptors in the murine epididymis, and because of the importance of the epididymidis in the regulation of sperm maturation, the identification of the transcripts regulated by androgens might facilitate the elucidation of the process of sperm maturation [21].

Many members of the beta-defensin family have shown exclusive expression in the epididymis. Except for the 6-cysteine domain, which is rich in positively charged amino acids, beta-defensins differ considerably in their amino acid sequences and target pathogen specificity. They form an important component of the epididymal innate immune system and provide protection against a broad range of pathogens that could invade the male reproductive tract, including E. coli, S. aureus and C. albicans [5-7]. Recent research has identified additional functions for beta-defensins that link the innate and adaptive immune responses. Beta-defensins cross-talk with the adaptive immune system by interacting with specific chemokine and toll-like receptors on immature dendritic cells and memory T cells resulting in the modulation of immunocompetent cell responses of the host [22].

Epididymal beta-defensins have evolved to perform functions other than immunoprotection, such as promoting sperm maturation, as suggested by the presence of several defensins on the sperm surface. However, little is known about the hormonal regulation, especially androgenic regulation, of the expression of beta-defensins.

In this study, androgen and AR regulation on caput epididymal beta-defensins was investigated. Beta-defensins exhibited different responses to androgen manipulation; most were regulated by androgen, some were not regulated by androgen or testicular factors, and one, Defb25, was regulated by testicular factors and not by androgen.

Among the androgen regulated beta-defensin genes, Defb20, 41, 42 and Spag11a were previously reported to be regulated by androgen [14-16]; our study further verified that these genes were direct targets of AR and had AR binding sites in the proximal promoter regions. Defb30 was previously shown to be regulated by testicular factors and not by androgens [16]. In our study, Defb30 was partially regulated by androgen, and it could be a direct target of AR. This discordance might be attributed to the sensitivity of different methods. The previous study used Northern blot to detect the expression of Defb30, whereas the current study used quantitative RT-PCR. Compared with quantitative RT-PCR, Northern blot has a low sensitivity. We reanalyzed the previous data and found a weak staining signal in the androgen replacement mouse epididymis compared with the castrated mouse epididymis, although the authors ignored it.

Jalkanen et al. reported that Defb42 was partially regulated by androgen [15], whereas Oh et al. reported that Defb42 was regulated by testicular factors and not by androgen [16]. Our results demonstrated that Defb42 was regulated by androgens. To investigate this discrepancy, we reanalyzed the data from Oh et al. and determined that the Defb42 in their paper should be classified as Defb48 based on sequence alignment. Oh et al. also reported that Defb44 was regulated by androgen; however, in that paper, Defb44 should be classified as Defb42 based on the sequence alignment.

AR regulates the expression of target genes by binding to AREs in the genome. Our previous work has identified genome-wide AR binding sites in the mouse epididymis, and in this study, we screened and identified ARBSs associated with androgen-regulated beta-defensins. These sites were located in the proximal promoters or intronic regions of target genes, indicating that their expression is directly regulated by AR. Consistent with our expectations, potential ARE motifs were identified in the majority of ARBSs, and these motifs conformed to consensus sequences that were highly similar to the established ARE in the MatBase database. However, no potential AREs were identified in the two ARBSs associated with Defb20 and Defb30/42, suggesting AR regulation by a tethering mechanism. AR regulation occurs without the direct binding of AR to AREs and likely through tethered associations of the receptor with its target genes [23]. For the ARBS associated with Defb30/42, an AP-2 motif was found in the binding summit. AP-2 has been reported to be an AR coregulator in the mouse caput epididymidis [14,24]. Thus, AR might be recruited to the ARBS by interacting with AP-2. Another possibility that could not be excluded is that there could be AREs that do not conform to the consensus sequence in the ARBSs associated with Defb20 and Defb30/42. For example, our previous data showed that noncanonical or half-site-like AREs were enriched in 38.3% of ARBSs without canonical AREs in the mouse epididymis. Those genes that changed expression, although were not bound by an AR, might be indirect AR target genes or might be regulated via long-range regulation (binding outside our defined window).

We tested the androgen responsiveness of Defb22, which is the mouse orthologue of human Defb126, and determined that it was partially regulated by androgen. Our ChIP-qPCR data identified an AR binding site in the distal promoter region, suggesting that Defb22 was a direct target of AR. Among the 9 genes deleted by Zhou et al. [11], four (Defb1, 13, 15, and 35) were tested in our study. Defb15 was partially regulated by androgen, whereas the three other defensins (Defb1, 13, 35) were not regulated by androgen.

The present study provides novel insights into the mechanisms of androgen regulation on epididymal beta-defensins. Sperm motility and fertilization capacity have been reported to be regulated by androgen [20]. Some AR-target beta-defensins, such as Spag11a, Defb22 and Defb15, might be involved in this regulatory process. Further studies are needed to clarify the functions of all of the AR target beta-defensins to help elucidate the molecular mechanism of sperm maturation and fertility.

## Conclusions

The current study demonstrated that 23 beta-defensins from caput epididymis showed different responsiveness to androgen, and twelve of them were identified to be directly regulated by androgen receptor. These results shed new light on the mechanisms of androgen/AR regulation on epididymal gene expression.

## Abbreviations

AR: Androgen receptor; ARE: Androgen response element; ARBS: Androgen receptor binding site; ChIP: Chromatin immunoprecipitation; MGI: Mouse genome informatics; RIA: Radioimmunoassay; TP: Testosterone propionate.

## Competing interests

The authors declare that they have no competing interests.

## Authors’ contributions

SGH performed quantitative RT-PCR, data analysis and wrote the manuscript. MZ performed literature research and statistical analysis. GXY performed ChIP assays while WBM and XQL constructed the mouse castration model. QLZ performed bioinformatics analysis. ZJC and YS designed the study and took part in the manuscript preparation. All authors read and approved the final manuscript.

## Supplementary Material

Additional file 1: Table S1 Primers for quantitative RT-PCR. Click here for file

Additional file 2: Table S2 Primers for ChIP-PCR/qPCR. Click here for file

Additional file 3: Figure S1 The potential ARE sequence (upper panel) in ARBSs associated with androgen-regulated beta-defensins identified by Weblogo is very similar to the ARE in the MatBase database (bottom panel). Click here for file
