# Supplementary material for: Androgenic regulation of beta-defensins in the mouse epididymis
Source: Reprod Biol Endocrinol. 2014 Aug 7;12:76. doi: 10.1186/1477-7827-12-76 (PMC4127520; doi:10.1186/1477-7827-12-76)
Supplement: Additional file 1: Table S1 — Primers for quantitative RT-PCR. [file 1477-7827-12-76-S1.doc]

**Supplemental Table 1 Primers for quantitative** RT-PCR

| **defensin** | **Forward primer (5'to 3')** | **Reverse primer (5'to 3')** | **PCR product** |
| --- | --- | --- | --- |
| **Defb1** | CTTTTCTCCCAGATGGAGCCA | GGGCTTATCTGGTTTACAGGTTC | 153 |
| **Defb12** | ATGGCTCTGAGCAGAGAGGTGT | CGCAATCTTCTCACTCTCTATGC | 192 |
| **Defb13** | TCTTCTCATTCAACTTTACCCAGC | GGTCCCAATCTTTTGCTCGA | 115 |
| **Defb15** | aatgcagcagggttaatggga | tgaagaactcgttgctctctgcc | 69 |
| **Defb18** | GTTTGGCATCATCCTGATGGT | CAGGGCTCAGTGTGACAGTAGTTAT | 174 |
| **Defb19** | ATCCTATCCTTCAATGCATGGG | CAGTGCTTCTCGTAAGACCAGTTAG | 172 |
| **Defb20** | CCTTGTTTTGCTGTTTGTGGC | CTTGTGCTTTTTGTTCTCCAGCT | 169 |
| **Defb25** | AGTCATGTACCACCAGGAAGCA | GAAGGCTTGAAAGAATAGGACAGG | 146 |
| **Defb29** | TACTTCATGACTGTGGTGGTCGT | CTGGATCTCATATTTCTGGCAGG | 153 |
| **Defb30** | CTTTGTCTTGCTCTCCTATGTTCC | CTCTAAGCAGCACAACGACTGAAT | 166 |
| **Defb34** | gcaggattaatgggagatgcacagcatcttgt | cgaagaacttgttgctctctgctgg | 65 |
| **Defb35** | TTTTGCAGCTCTTTCCTGGC | CCTCTCTCGACAGCAGAAGAAGT | 143 |
| **Defb37** | TCCTCTCTCTATCCAACTTCCAGAA | CACTTGCCTTTTCCTTCATAACAG | 145 |
| **Defb39** | ATGAAGATCTCCTACTTTCTGCTGC | CAACACTTGCCTCTTCTGTCATAAC | 176 |
| **Defb40** | ATCATGCTTTCAGATCAACCCAG | GGTACACATGATTTCTGGCAAAC | 176 |
| **Defb41** | TCTTTATTCTGCTCTTTGGGGC | GGTTCCTCCATCTGATACAGTATCC | 156 |
| **Defb42** | CATGCTTACTGTTCATCCTGGTG | TGTTACAGTAGGCAATCCATACCC | 150 |
| **Defb45** | AGACTGCGGTGTTAACTATGGCT | GTTTGCCATTCCAGCAGAAGA | 144 |
| **Defb51** | CTCTTTGCCGTCTTCCAGGTA | GCAGACAGTGAAAGCTCTGCA | 147 |
| **Defb52** | CCTTCATCAATGCCTTCATCAA | AGGTCATGGTTCTCATACAGCAG | 144 |
| **spag11a** | GTTACTCTTTGCTGTCTTCTTCTGC | GGAGCAGATATCCCCTTTTCTCT | 148 |
| **spag11b/c** | GCCAGTCTACTTTTTGCAGCC | TTGGTTCAGGCTCGTGGTAAG | 193 |
| **Defb22** | ACTCTTGTGATCATTATGTTCCTGG | CACAGTTTTCCTATGGGACATTTG | 155 |
