# Supplementary material for: Androgenic regulation of beta-defensins in the mouse epididymis
Source: Reprod Biol Endocrinol. 2014 Aug 7;12:76. doi: 10.1186/1477-7827-12-76 (PMC4127520; doi:10.1186/1477-7827-12-76)
Supplement: Additional file 2: Table S2 — Primers for ChIP-PCR/qPCR. [file 1477-7827-12-76-S2.doc]

**S**upplemental Table 2 Primers for ChIP-PCR/qPCR

| **defensin** | **Forward primer (5'to 3')** | **Reverse primer (5'to 3')** | **PCR product** |
| --- | --- | --- | --- |
| **Defb15** | TCCCTTGACTTTTACAGTAGGCTCT | GGAGAAGTGCTGGATTCTTACAAA | 152 |
| **Defb18** | CAGGAACATTTCGCTTGGAGTA | GTAGTGCTAAGATCTTGAAGGTTGC | 140 |
| **Defb19-1** | AAGCTACCTCCATCAACTCTCTCTA | GATTGACTTGGTTACTGGAATGTG | 179 |
| **Defb19-2** | CTGTGGGAGACAAGATGAGAACAA | ACAATAGTTGCCCAGATTGGAAG | 129 |
| **Defb20** | ATCTCGTTATCTTCTCCAGCCTACT | TCCCGGAGCTACTGTCAAATTAC | 124 |
| **Defb30** | GTGTTACAATGTCTGTGGGAAGAAT | TAAGACTTCATGCTCTCATACCCTG | 122 |
| **Defb34** | AACAGCAACACACTGTAGGTTGAG | TATACGCTTCTCCAGAGCAAACA | 127 |
| **Defb37** | TCTTGACCAAAGCAAAGTCAGC | GGATCGCAGACTATCTCAAATGTG | 115 |
| **Defb39** | GAGCTTCAAAGAAGCAAGGCTT | TGGGAAAAAGCTATCAGGAAGC | 128 |
| **Defb41** | TGAGGCTGAGAATCCAAGAACAC | TGAAGTAGCCATGTGTTTGGGT | 117 |
| **spag11a** | TCGGCAACTTTCTGTACTTTTACG | TCTCCAGCTTGACGACAATGATC | 100 |
| **Defb22** | TATGCTTCAGGTCTTCTCATACAGC | TGAAGTGCAGGGATGTTACTCAG | 143 |
