# Supplementary figures and images for: Androgenic regulation of beta-defensins in the mouse epididymis
Source: Reprod Biol Endocrinol. 2014 Aug 7;12:76. doi: 10.1186/1477-7827-12-76 (PMC4127520; doi:10.1186/1477-7827-12-76)

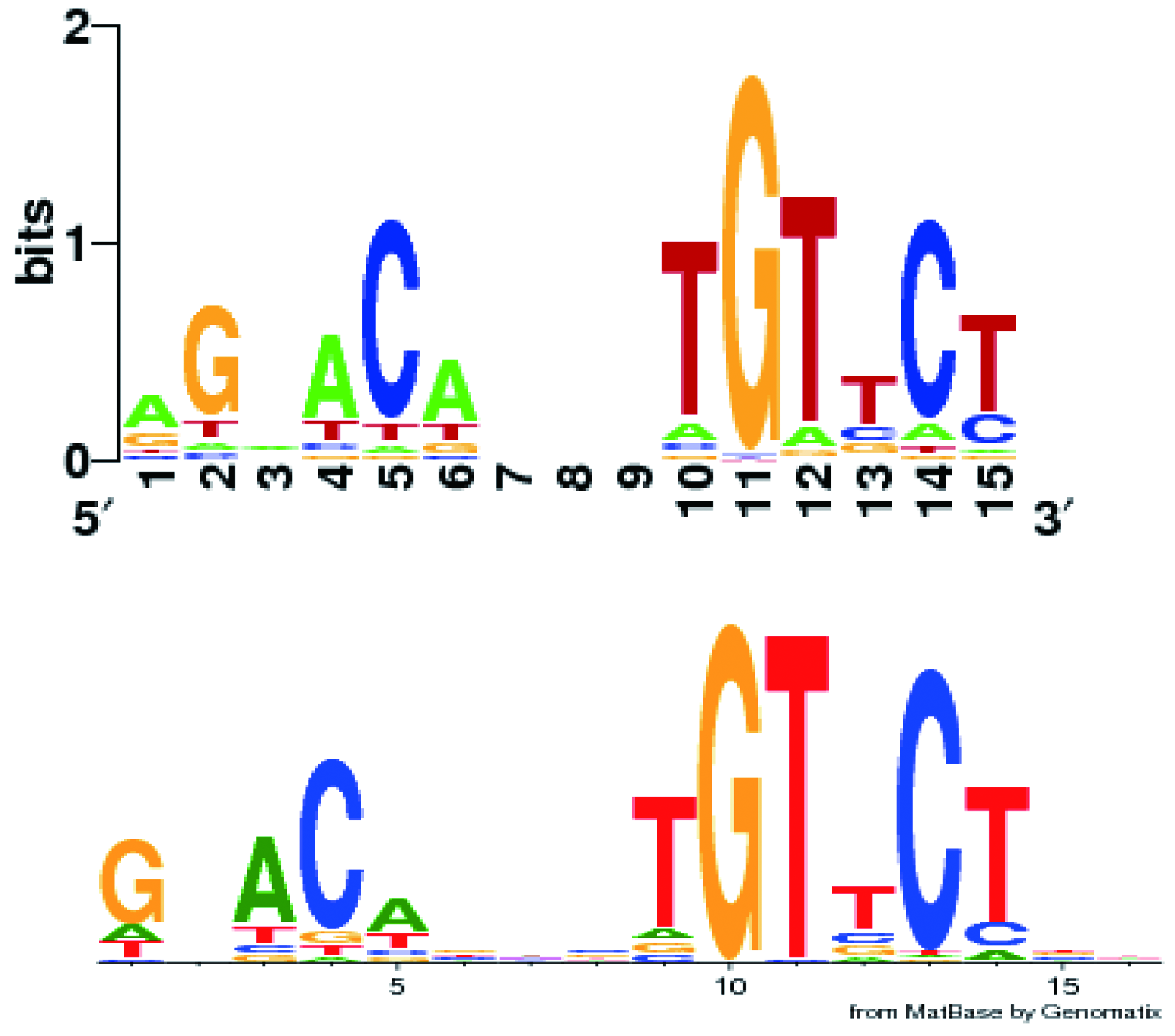

Supplement: Additional file 3: Figure S1 — The potential ARE sequence (upper panel) in ARBSs associated with androgen-regulated beta-defensins identified by Weblogo is very similar to the ARE in the MatBase database (bottom panel). [file 1477-7827-12-76-S3.tiff]
